# RNA-seq analyses of changes in the *Anopheles gambiae* transcriptome associated with resistance to pyrethroids in Kenya: identification of candidate-resistance genes and candidate-resistance SNPs

**DOI:** 10.1186/s13071-015-1083-z

**Published:** 2015-09-17

**Authors:** Mariangela Bonizzoni, Eric Ochomo, William Augustine Dunn, Monica Britton, Yaw Afrane, Guofa Zhou, Joshua Hartsel, Ming-Chieh Lee, Jiabao Xu, Andrew Githeko, Joseph Fass, Guiyun Yan

**Affiliations:** Program in Public Health, University of California, Irvine, CA USA; Department of Biology and Biotechnology, University of Pavia, Pavia, Italy; Centre for Global health Research, Kenya Medical Research Institute, Kisumu, Kenya; Department of Ecology and Evolutionary Biology, Yale University, New Haven, CT USA; Bioinformatics Core of the UC Davis Genome Center, University of California, Davis, CA 95616 USA; School of Health Sciences, Jaramogi Oginga Odinga University of Science and Technology, Bondo, Kenya; Delta-9 Technologies, San Diego, CA 92101 USA

**Keywords:** *Anopheles gambiae*, insecticide resistance, RNA-seq, metabolic detoxification

## Abstract

**Background:**

The extensive use of pyrethroids for control of malaria vectors, driven by their cost, efficacy and safety, has led to widespread resistance. To favor their sustainable use, the World Health Organization (WHO) formulated an insecticide resistance management plan, which includes the identification of the mechanisms of resistance and resistance surveillance. Recognized physiological mechanisms of resistance include target site mutations in the *para* voltage-gated sodium channel, metabolic detoxification and penetration resistance. Such understanding of resistance mechanisms has allowed the development of resistance monitoring tools, including genotyping of the *kdr* mutation L1014F/S in the *para* gene.

**Methods:**

The sequence-based technique RNA-seq was applied to study changes in the transcriptome of deltamethrin-resistant and -susceptible *Anopheles gambiae* mosquitoes from the Western Province of Kenya. The resulting gene expression profiles were compared to data in the most recent literature to derive a list of candidate resistance genes. RNA-seq data were analyzed also to identify sequence polymorphisms linked to resistance.

**Results:**

A total of five candidate-resistance genes (AGAP04177, AGAP004572, AGAP008840, AGAP007530 and AGAP013036) were identified with altered expression between resistant and susceptible mosquitoes from West and East Africa. A change from G to C at position 36043997 of chromosome 3R resulting in A101G of the sulfotransferase gene AGAP009551 was significantly associated with the resistance phenotype (odds ratio: 5.10). The *kdr* L1014S mutation was detected at similar frequencies in both phenotypically resistant and susceptible mosquitoes, suggesting it is no longer fully predictive of the resistant phenotype.

**Conclusions:**

Overall, these results support the conclusion that resistance to pyrethroids is a complex and evolving phenotype, dependent on multiple gene functions including, but not limited to, metabolic detoxification. Functional convergence among metabolic detoxification genes may exist, with the role of each gene being modulated by the life history and selection pressure on mosquito populations. As a consequence, biochemical assays that quantify overall enzyme activity may be a more suitable method for predicting metabolic resistance than gene-based assays.

**Electronic supplementary material:**

The online version of this article (doi:10.1186/s13071-015-1083-z) contains supplementary material, which is available to authorized users.

## Background

Over the past decade, several initiatives including the Global Fund, the President’s Malaria Initiative, private foundations and national governments supported a massive scale-up of antimalarial interventions in Africa [1, 2]. These control programs targeted malaria vectors, through insecticide-treated nets (ITN) and indoor residual spraying (IRS), as well as human hosts by improving diagnosis and implementing artemisinin-combination treatments (ACT). As a result, the annual number of malaria-related deaths in sub-Saharan Africa decreased by 49 % between 2000 and 2012 [3]. However, malaria still kills more than half a million people a year [3] and weakening of malaria control programs could favor malaria resurgence [4]. As a consequence, the identification of elements that could threaten the sustainability of malaria control strategies is critical to continue the fight against this disease. Currently, the core strategies of vector control (IRS and ITN) rely on insecticides [5]. The World Health Organization (WHO) recommends the use of four classes of insecticides in IRS (pyrethroids, organochlorine, organophosphate and carbamate), while only pyrethroids are approved for use on ITNs [6]. Extensive use of insecticides imposes selection pressure on mosquito populations for increased resistance. Resistance to insecticides is widespread geographically in Africa and involves primarily, but is not limited to, pyrethroids [6–8]. As a response to this situation, the WHO formulated an action plan to support the sustainability of control programs focused on the use of insecticides [6]. The understanding of the mechanisms of insecticide resistance and the monitoring of resistance are two of the five pillars of this plan [6]. Historically, the identification of the mechanisms of resistance has been important for developing molecular monitoring tools of resistance [9].

One of the main malaria vectors in sub-Saharan Africa is *Anopheles gambiae sensu strictu* (hereafter referred as *An. gambiae*). *An. gambiae* exists as two molecular forms (M and S), emerging as distinct species, mainly due to reduced fitness of hybrids in nature [10, 11]. The S form, named *An. gambiae*, has the broadest distribution, occurring in West and East Sub-Saharan Africa; the M form, named *Anopheles coluzzii*, is primarily confined to West Africa, with the exception of northern Zimbabwe [10]. The same types of resistance mechanisms have been identified in *An. gambiae* and *An. coluzzii*, including behavioral and physiological resistance. Physiological resistance includes target site mutation, metabolic detoxification, and penetration resistance. The target site for pyrethroids is the *para* voltage-gated sodium channel [12]. Two alternative amino-acids changes at position 1014 of the *para* gene (L1014S and L1014F) have been associated with resistance [12, 13]. Originally detected in West (L1014F) and East (1014S) Africa, both mutations are now found across mosquito populations from sub-Saharan Africa, but at different frequencies. The L1014F mutation is rare in East Africa, but approaches fixation in many countries of West Africa; the L1014S allele has increased in frequency across all sub-Saharan Africa in the past decade [8, 9]. An additional mutation (N1575Y) was detected recently in *An. coluzzii* mosquitoes from Burkina Faso that harbored the L1014F mutation [14]. The N1575Y mutation is thought to either enhance resistance due to the L1014F mutation or compensate for its fitness costs [14]. The frequency of this mutation is unknown in *An. gambiae*.

Metabolic detoxification occurs when pyrethroids are catabolized or sequestered and eliminated before they reach the voltage-gated sodium ion channel [12]. Enzymes encoded by three large gene families (esterases, P450 mono-oxygenases [CYPs] and glutatione-*S* transferases [GSTs]) play important roles in insecticide detoxification [11, 15]. Gene-expression studies identified several detoxification genes over-expressed in resistant vs susceptible *An. gambiae* and *An. coluzzii* laboratory strains and wild populations, with the products of two genes (CYP6P3 [AGAP002865] and CYP6M2 [AGAP008212]) shown to be able to metabolize pyrethroids [16, 17]. A state of stress, manifested primarily through lipid peroxidation, is also seen upon insecticide exposure [18, 19]. Target site mutation and metabolic detoxification may co-occur with varying frequencies in natural populations and their relative contribution to resistance phenotype may be influenced by the intensity of vector control strategies and the breeding environments [20, 21]. Furthermore, other physiological mechanisms of resistance have been proposed, including thickening of the cuticle and modification of the digestive tract lining, which may help reduce insecticide penetration and absorption (penetration resistance) [8, 22]. Behavioral modifications, such as earlier biting and outdoor feeding, which results in mosquitoes avoiding insecticides, have also been recognized as important factors contributing to increased resistance (behavioral resistance) [23]. However, current data are insufficient to determine whether behavioral resistance traits are genetic or adaptive and the difficulty in accurately measuring mosquito behavior in the wild has limited the understanding of behavioral resistance [23].

We examined the gene expression profile of deltamethrin-resistant and -susceptible mosquitoes from the Western Province of Kenya by RNA-seq to further the understanding of resistance mechanisms and possibly characterize markers for resistance surveillance. The application of this sequence-based approach allowed us to interrogate absolute changes in transcript accumulation profiles and investigate single nucleotide polymorphisms (SNPs) that may be associated with insecticide resistance. A step-wise filtering approach, including comparison to literature data [24], was used to identify a total of five candidate resistance genes. Additionally, for SNPs, the *kdr* L1014S mutation was detected at similar frequencies in phenotypic pyrethroid-resistant and susceptible-mosquitoes and the non-synonymous change A101G in sulfotransferase gene AGAP009551 was found to be associated with the resistance phenotype.

## Methods

### Mosquito samples

**Fig. 1:**
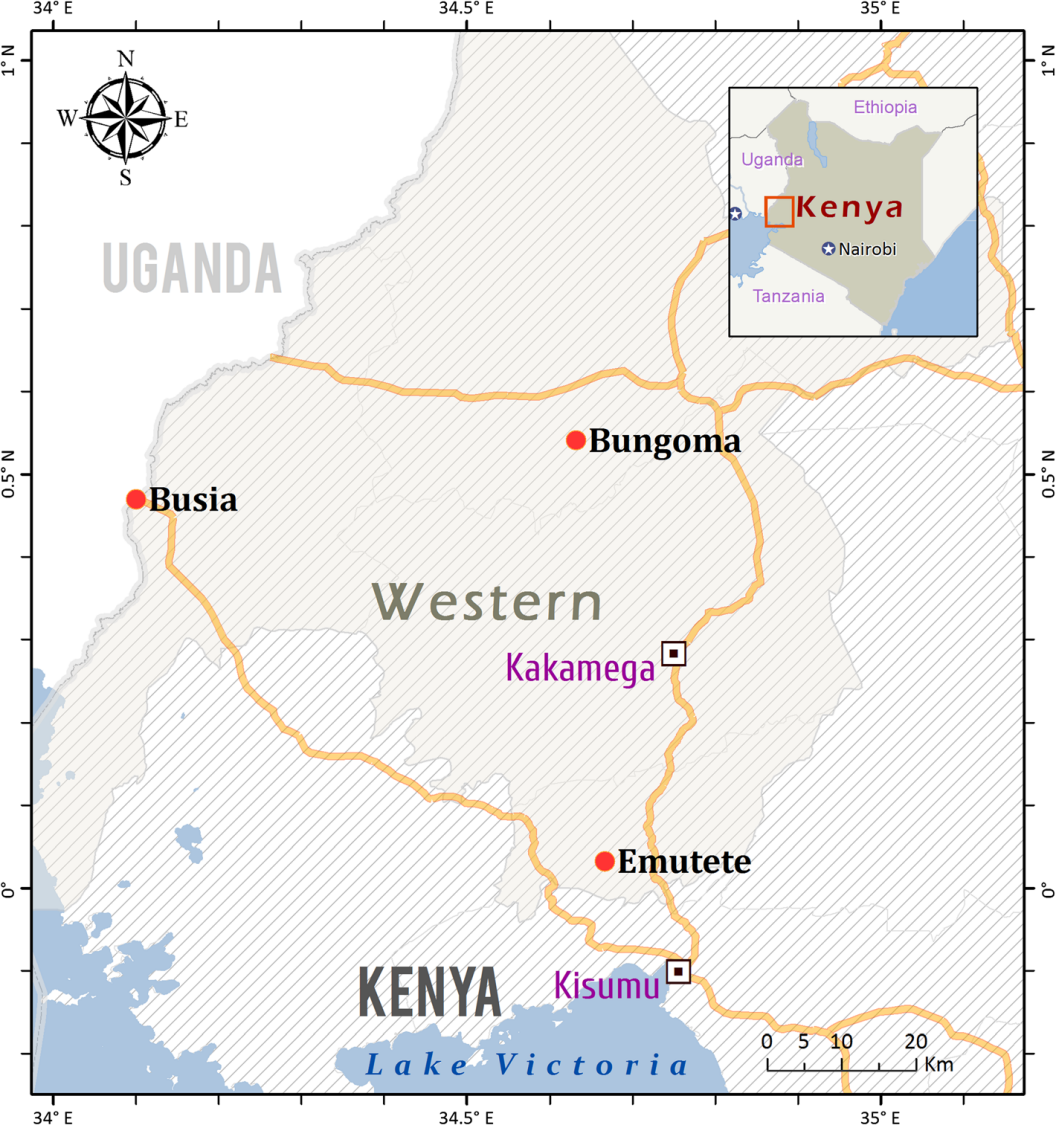
Map of the Western Province of Kenya. Localities in the Western Province of Kenya around which multiple larvae collections occurred are shown with a red circle. Kagamega and Kisumu are the capitals of the Western and Nyanza Provinces, respectively. Nearby countries are shown with a square and in purple. Main roads are in yellow

*Anopheles gambiae* larvae were collected in the Western Province of Kenya, in breeding sites around the localities of Bungoma, Busia and Emutete in 2012 (Fig. 1). Multiple mosquito samplings in different localities within the Western Province allows capturing the mosquito genetic variability of this region and makes gene expression analysis results more broadly applicable. A previous survey identified wide-spread resistance to deltamethrin in mosquitoes from this area, with mortality ranging between 66.5 and 78.7 % [25]. Larvae were transported to the insectary of the Centre for Global Health Research, Kenya Medical Research Institute (KEMRI) in Kisumu, and reared to adulthood for the resistance bioassay. The prevalent mosquito species in this area is *An. gambiae* [24]. Mosquitoes of the Kisumu strain also were used. The Kisumu strain originated in the early 1990s and is the pyrethroid susceptible standard strain [26].

### Phenotypic resistance to deltamethrin

Resistance to deltamethrin was tested by the standard WHO tube test [7] on single mosquitoes reared from field-collected larvae. Mosquitoes alive 24 h after the 60-min insecticide-exposure time were classified as resistant. Susceptible mosquitoes were those, which were knocked-down early after the insecticide exposure. Knocked-down mosquitoes were observed for 2–3 min, and the tubes were moved gently to confirm mosquitoes showed no signs of recovery; morphological signs of distress were also checked (i.e. loss of legs) before considering mosquitoes as susceptible. Upon collection, mosquitoes were stored in RNA-later (Ambion) to avoid RNA degradation. This strategy for collecting resistant- and susceptible-mosquitoes allows sampling mosquitoes from the same locality and minimizes the impact of heterogeneous genetic background and environmental conditions on gene accumulation profiles. Additionally, early knock-down has already been shown to be a valid approximation for susceptibility [25, 27, 28]. However, this phenotyping method will not allow differentiation between insecticide-induced and constitutive differential expression between resistant- and susceptible- mosquitoes. Constitutive differential expression will be investigated by comparing data from field-collected mosquitoes and mosquitoes of the susceptible Kisumu strain, which is expected to be highly inbred [26]. The WHO tube tests were conducted at the same hours of the day on all experiments to avoid influence of the circadian clock on transcript accumulation profiles [29].

### Nucleic acids extractions

DNA was extracted from mosquito legs using the Fast Tissue-to-PCR kit (Fermentas). Total RNA was isolated from single mosquitoes using Trizol (Invitrogen) [30]. RNA concentration was measured by NanoDrop. RNA quality was analyzed on an Agilent 2100 Bioanalyzer.

### Species discrimination between *An. gambiae* and *An. arabiensis* and genotyping of the *para*-sodium channel gene

Each deltamethrin-phenotyped mosquito was identified as *An. gambiae* or *An. arabiensis* by amplifying the species-specific rDNA [31]. Codons 1014 and 1575 of *para* sodium channel gene (AGAP004707) were analyzed on a total of 324 *An. gambiae* mosquitoes by direct sequencing of the fragments obtained by the polymerase chain reaction (PCR) with primers Adg1 and Adg2 [13] and 1575-F (5′ TAAACAGCCTATACGGGAAACG) and 1575-R (5′ CGAGGAATTGCCTTTAGAGGTTTCT), respectively.

### RNA-seq library preparation and sequencing

A total of nine RNA-seq libraries were prepared from pooled RNA of 12 mosquitoes each (Table 1). Illumina paired-end RNA-seq libraries were prepared following a standard procedure and sequenced for 100 bp from each end on an Illumina HiSeq2500 at the DNA Technologies and Expression Analysis Core at the Genome Center of the University of California Davis [32]. Raw sequencing data have been deposited at NCBI’s Sequence Read Archine (SRA) under study accession number SRP052073.

**Table 1:** RNA-seq sample summary

Origin	RNA pool^2^	Total aligned reads^3^
Western Province, Kenya	Resistant_0	144,917,184
	Resisistant_1	154,463,158
	Resistant_2	138,266,938
	Resistant_3	152,222,775
	Susceptible_0	139,954,740
	Susceptible_1	146,021,244
	Susceptible_2	124,918,668
	Susceptible_3	128,348,124
Kisumu strain	Kisumu_0	127,550,869

^2^The total RNA from 12 mosquitoes was pooled in equal molarity after having verified its quality

^3^Total number of million reads aligned to the *Anopheles gambiae* genome (AgamP3.7 gene set)

### Data analyses

Raw read quality and contamination was evaluated using the Bioconductor package qrqc [33]. Scythe v.0.990 and Sickle v.1.200 [34] were used for Illumina adapter and quality-based trimming. Differential expression analyses followed the Tuxedo pipeline [35], which was run in Blacktie [36], using the VectorBase *An. gambiae* assembly P3 (and associated annotation AgamP3.7). Relationships among conditions and replicates were explored with Multiple Dimensional Scaling (MDS). MDS was computed using CummeRbund, a program within the Tuxedo pipeline [37]. MDS is a linear transformation method of variance stabilized count where the directions that maximize the separation (or discrimination) between the different samples are visualized. Transcript functional annotation was conducted using the biomart function in VectorBase [38] and AnoXcell [39]. Functional enrichment analyses was done using g: Profiler [40].

SNPs were called with Freebayes v. 0.9.4 and SnpEff [41, 42], following a previously described pipeline [43]. Prior to variant finding, multiple mapping reads (those with a mapping quality of zero) were removed from the Tophat output. Programs for estimating allele frequency from DNA-seq data of large pooled samples (>50) have been developed [43] and used successfully on RNA-seq data from highly inbred and not biologically-challenged samples [44, 45]. In our case, we expect RNA-seq data to include population variation and reflect differential expression of transcripts because we are using mosquitoes from the field and our experimental design compares mosquitoes of two different phenotypes (resistant and susceptible). Differential coverage across genes will result in unbalanced pool composition and biased allele frequency estimates when treating RNA-seq data as DNA-seq [43], as a consequence, we abstain from estimating SNP allele frequency using RNA-seq data through a DNA-seq-focused pipeline.

### qRT-PCR validation of RNA-seq data

The accumulation levels of 18 transcripts were analyzed by quantitative real-time reverse transcription PCR (qRT-PCR). Specifically, cDNA was prepared using SuperScript III (Invitrogen) and random primers from pooled RNA of 3 resistant or 3 susceptible mosquitoes, including a total of 27 resistant and 27 susceptible mosquitoes. qRT-PCR reactions were run and analyzed as described previously using the S7 ribosomal protein gene as internal control [25, 46]. RNA from different phenotyped mosquitoes was used for RNA-seq library preparation and qRT-PCR experiments, providing for biological replicates. The Pearson correlation coefficient was calculated between fold changes in transcript accumulation levels between resistant and susceptible mosquitoes as obtained by qRT-PCR and RNA-seq, respectively [47].

### Genotyping of candidate resistance genes and association with the resistance phenotype

The coding sequences of 39 genes harboring SNPs identified from RNA-seq libraries were analyzed in individual resistant and susceptible mosquitoes (Additional file 1). Specifically, genomic DNA was extracted from 54 resistant and 54 susceptible mosquitoes using the Fast Tissue-to-PCR kit (Fermentas). Genomic DNA was used as template in a PCR reaction with 11.5 μl of Master mix (Fermentas) and 10 μM of each forward and reverse primers (Additional file 1). PCR reactions were run in a MyCycler (Biorad) under the following conditions: 94 °C for 3 min followed by 40 cycles of 94 °C for 30 s, 50–54 °C for 45 s and 72° for 45–90 s. PCR products were sequenced directly, using the standard Sanger method [48]. The odds ratio test was applied to determine if the odds of each analyzed SNP differed significantly between resistant and susceptible mosquitoes [49].

## Results

### para gene polymorphism and “deltamethrin -resistant or -susceptible” phenotype

A total of 324 *An. gambiae* adults were genotyped at the *para* gene after their phenotype was established as “resistant” or “susceptible” to deltamethrin using the standard WHO tube test [7]. All resistant and >90 % of the susceptible mosquitoes were homozygous for the codon encoding for serine at position 1014 of the *para* gene. One susceptible and one resistant mosquito were homozygous for the codon encoding for phenylalanine at position 1014. The N1575Y mutation was not detected in any tested mosquito.

### RNA-seq libraries of deltamethrin-resistant and susceptible mosquitoes: quality-control

**Fig. 2:**
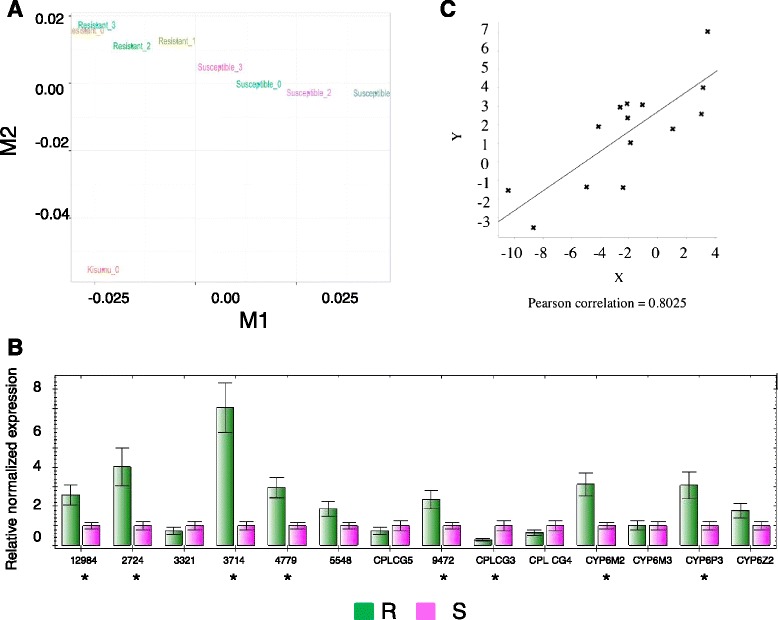
Quality control of RNA-seq data. **a** multidimensional scaling (MDS) plot showing variation among RNA-seq libraries. RNA-seq libraries from resistant, susceptible and Kisumu mosquitoes. **b** Results of qRT-PCR. The level of expression of 14 genes was measured by qRT-PCR from different resistant and susceptible mosquitoes than those used for RNA-seq. An axterix indicate genes significantly differentially expressed between resistant (green) and susceptible (pink) mosquitoes. **c** Pearson correlation between fold-changes in gene expression between resistant and susceptible mosquitoes as determined by qRT-PCR (X-axe) and RNA-seq (Y-axe)

Paired-end Illumina RNA-seq libraries were constructed and 100 bp were sequenced from each end from four RNA pools of 12 resistant or susceptible mosquitoes from the Western Province of Kenya and one pool of 12 mosquitoes of the susceptible Kisumu strain. The total number of reads per library aligned to the *An. gambiae* genome (AgamP3.7 gene set) ranged from 124,918,668 to 154,463,158 and showed no significant differences across samples (Table 1, Additional file 2). Variation in gene expression among libraries was visualized by MDS, which resulted in suggested two distinct clusters for the resistant and susceptible replicate samples (Fig. 2a). The sample consisting of pooled mosquitoes of the Kisumu strain was markedly distant from either of the two other groups (Fig. 2a). The greater distance between field mosquitoes and mosquitoes of the Kisumu strain may have resulted from different life history (e.g., exposure to insecticides and fluctuating temperature) or variation in larval rearing conditions. Distance among libraries within each cluster reflects variations among samples. Variations in gene expression profiles, which are suggestive of a broad spectrum of transcription within “resistant” and “susceptible” phenotypes, was expected because the WHO tube assay used to phenotype mosquitoes is a binary test that discriminates based on a 60 min exposure to a standard insecticide-discrimination dose (0.05 % deltamethrin). Finally, we compared gene expression levels estimated from RNA-seq and qRT-PCR for 14 genes. Eight genes (AGAP012984, AGAP002724, AGAP003714, AGAP004779, AGAP009472, CPLCG3 [AGAP008446], CYP6M2 [AGAP008212] and CYP6P3 [AGAP002865] were found to be significantly differentially expressed between resistant and susceptible mosquitoes by qRT-PCR and a significant positive correlation (Pearson correlation = 0.8025, *p* < 0.01) was found between expression values as detected by qRT-PCR and RNA-seq (Fig. 1b).

### Differential expression of genes between field-caught resistant and susceptible mosquitoes

**Fig. 3:**
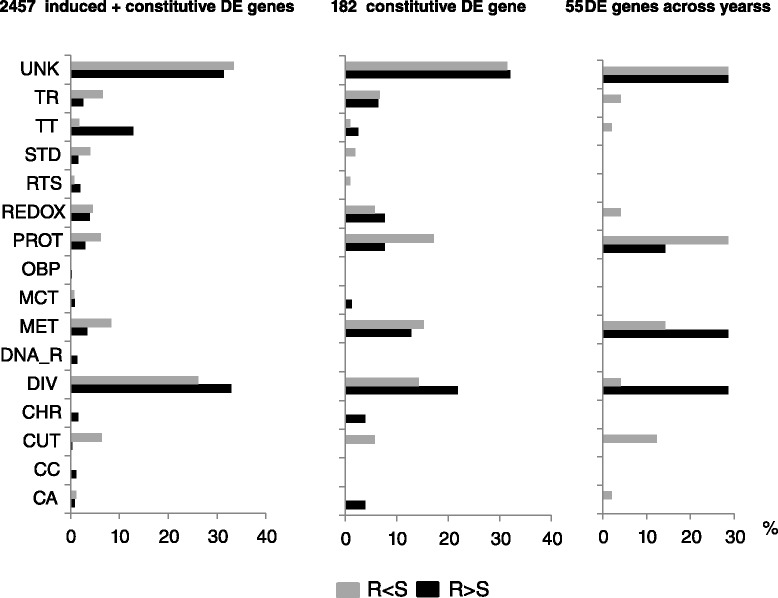
Functional classifications of candidate-resistance genes. Genes expressed significantly more (R > S) or less (R < S) in resistant versus susceptible mosquitoes were classified based on their functions. A percentage was attributed to each function based on the total number of genes considered. Functional abbreviation is as follows: UNK (Unknown); TR (transport); TT (transcription and translation); STD (signal transduction); RTS (response to stress); REDOX (oxido-reduction processes); PROT (proteolysis); OBP (odorant binding proteins); MCT (microtubule-associated movement); MET (metabolism); DNA_R (DNA repair); DIV (diverse functions); CHR (chromosome or chromatin-related functions); CUT (cuticule); CC (cell cycle); CA (catalytic activity)

Candidate resistance genes were identified based on the assumption that they are significantly differentially expressed (DE) between resistant- and susceptible-mosquitoes (Additional file 3). A total of 2457 genes (18.36 % of 13361 genes tested) were found to be significantly DE (Additional file 4). Gene functional annotation revealed an enrichment in transcription-related functions, RNA processing, regulation of metabolic processes, chromatin organization, protein digestion and cellular response to stress among the 1373 genes more highly expressed in resistant than susceptible mosquitoes. The 1083 genes overexpressed in susceptible vs. resistant mosquitoes included genes associated with signal transduction, transport and proteolysis and were enriched in carbohydrate metabolic processes and cuticle (Fig. 3).

#### Detoxification genes

Thirty-nine detoxification genes were significantly DE, with 12 genes (HPX15 [AGAP013327], HPX7 [AGAP004036], HPX15 (AGAP013327), CYP304B1 [AGAP003066], CYP306A1 [AGAP004888], CYP315A1 [AGAP000284], CYP4C26 [AGAP000192], CYP4C27 [AGAP009246), CYP6M2, CYP6N1 [AGAP008210], CYP9J3 [AGAP012291], COEAE2G [AGAP006723]) showing more than 2 fold differential expression (Additional file 5). Among the detoxification genes previously associated with insecticide resistance [15, 16, 24, 50–52], CYPM2, CYP9J15 (AGAP012296), GSTT2 (AGAP009016) and GSTE5 (AGAP009192) were found to be 2.12, 1.78, 1.57 and 1.54 fold expressed more in resistant than susceptible mosquitoes, respectively; while CYP325C2 (AGAP002205) was expressed 2.23 fold more in susceptible than resistant mosquitoes, contrary to what was recently detected in *An. coluzzii* mosquitoes from West Africa [24].

#### Cuticular protein genes

A total of 64 cuticular protein genes were significantly DE (Additional file 4). The majority (95.31 %) of these genes was more highly expressed in susceptible than resistant mosquitoes, a trend that is consistent with previous results [18, 25]. The RNA-seq based lower expression in resistant than susceptible mosquitoes of two cuticular protein genes (CPLG3 [AGAP008446] and CPLG4 [AGAP008447]), previously likned to insecticide resistance in *An. gambiae*/*An. coluzzii* mosquitoes from West Africa [46, 53], was lower in resistant than susceptible mosquitoes in the RNA-seq analysis. This was confirmed by qRT-PCR on independent samples of phenotypic resistant and susceptible mosquitoes (Fig. 1c).

### Comparison with the Kisumu susceptible strain

To account for induction of gene expression during the insecticide exposure bioassay, we filtered the candidate-resistance genes by analyzing their expression profiles in mosquitoes of the Kisumu strain. We assumed constitutive candidate-resistance genes are significantly DE between field-resistant mosquitoes and mosquitoes of the Kisumu strain, but not between field-susceptible and Kisumu mosquitoes because resistance to insecticide is considered a pre-adaptive phenotype [54]. A total of 702 genes were significantly DE between resistant mosquitoes and mosquitoes of the Kisumu strain, while 12467 genes (93.30 % of the total number of tested genes teste) were not DE between susceptible mosquitoes from the field and from the Kisumu strain. This filtering approach reduced the number of candidate-resistance genes to 182 (Additional file 4), including 105 genes expressed more highly in susceptible mosquitoes and enriched in functions such as proteolysis, organic acid metabolic processes, transport and cuticle; and 78 genes expressed more highly in resistant mosquitoes and functionally related to endopeptidase activity, cytochrome P450s and nucleotide binding (Fig. 3).

### Comparison to previously detected candidate-resistance genes

We compared the 182 DE genes with DE genes previously detected from mosquitoes from Emutete, a rural town in the Western Province of Kenya, in 2010 [25]. A total of 55 common DE genes were identified, including seven overexpressed in resistant versus susceptible mosquitoes and linked to transferase activity and metabolic/detoxification processes (Additional file 5). Genes expressed more highly in susceptible than resistant mosquitoes were enriched in functions such as proteolysis and cuticle and included HPX2 (AGAP009033) and six cuticle protein encoding genes.

A list of 295 candidate resistance genes has been recently identified in *An. coluzzii* mosquitoes from Burkina Faso and Côte d’Ivoire by comparing deltamethrin-resistant field-derived mosquitoes and mosquitoes of two susceptible strains [24]. When a similar comparison is done across our RNA-seq data, a total of 21 candidate resistance genes was identified (Additional file 6), including two genes (AGAP004177, AGAP004572) mapping within a QTL previously linked to resistance to pyrethroid [55] and associated with RNA methylation and lipid metabolism, respectively (Additional file 6). These two genes and three others (AGAP013036, AGAP007530 and AGAP008840) are also DE when considering the comparison to susceptible mosquitoes from Western Kenya (Table 2). qRT-PCR analyses confirmed changes in expression profile as detected by RNA-seq for AGAP013036, AGAP008840 and AGAP004177; AGAP004572 showed higher expression in resistant versus susceptible samples. Within samples variation tended to be large, suggesting none of these genes has a major effect, but indirect and/or additive effects on the resistant phenotype are more probable (Additional file 7).

**Table 2:** Candidate resistance genes

			FPKM					
Gene	Chr.	Band	R	S	Kis.	R/S	R/K	Go term
AGAP004177	2R	18B	9.64	5.59	3.79	1.72	2.54	RNA methylation
AGAP004572	2R	19C	7.68	12.75	21.79	−1.66	−2.84	lipid metabolism
AGAP007530	2 L	28B	8.93	23.34	23.33	−2.61	−2.64	proteolysis
AGAP008840	3R	32A	7.68	21.29	23.56	−2.77	−3.07	protein binding
AGAP013036	2R	38A	95.37	60.82	17.59	1.57	5.39	cellular protein

### SNPs identified in RNA-seq libraries

#### SNPs in the pyrethroid target site

RNA-seq analysis of the *para* gene confirmed the presence and absence of the L1014S and N1575Y mutations, respectively. An additional change from A to G was identified at nucleotide position 2391280 in the 9^th^ exon of the *para* gene, leading to a change from lysine [K] to arginine [R]) at amino acid position 419, based on the *Musca domestica* Vss1 gene nomenclature [Accession N. AAB47604] (Additional file 8). The *para* gene codes for a protein with four homologous domains, each composed of six segments. Both the 419 and the 1014 mutations lie in the 6^th^ segment of the first and second domains, respectively [56]. While various mutations at position 1014 are wide-spread in insects, this is the first report of a non-synonymous mutation at position 419. Genotyping data on 96 Kenyan mosquitoes found the K419R mutation only in insecticide-susceptible mosquitoes.

#### SNPs in other genes: non-synonymous SNP potentially associated with insecticide-resistance in the sulfotransferase encoding gene AGAP009551

We searched for SNPs across all genes, including those not DE between resistant and susceptible mosquitoes, and identified 310 genes with SNP coverage only in resistant mosquitoes; in 102 of these genes susceptible mosquitoes showed coverage only for the not wild-type mutant (reference) nucleotide base (Additional file 9). The majority (92.26 %) of SNPs was associated with non-synonymous mutations, followed by start gain/loss (7.42 %) and splice acceptor/donors (0.32 %) (Additional file 9). A total of 39 SNPs were chosen for further genotyping in single mosquitoes based on: 1) their location within previously identified pyrethroid resistance QTLs [55], or 2) RNA-seq coverage (Additional files 1 and 9). In general, SNPs associated with pyrethroid resistance were rare. We found a change from G to C at position 36043997 of chromosome 3R that results in an Alanine to Glycine substitution at codon 1010 of AGAP009551, and was significantly associated with the resistance phenotype (odds ratio [95 %]: 5.10 [1.30–19.99]) (Additional file 8).

## Discussion

In this study, we compared the transcriptome of pyrethroid-resistant and susceptible *An. gambiae* mosquitoes from Western Kenya and we analyzed results using a step-wise approach starting from comparison among local mosquitoes and finishing with a comparison across *An. gambiae* and *An. coluzzii* originating from different ecosystems. We identified 5 genes consistently DE between resistant and susceptible *An gambiae*/*An. coluzzii* across Sub-Saharan Africa and 1 SNP strongly associated with the resistance phenotype.

### Markers for resistance surveillance

WHO standard bioassays are generally applied to assess phenotypic resistance of mosquito populations [7]. However, these methods require a large number of field-caught mosquitoes and variation in the age and physiological conditions of the specimens can affect the consistency of bioassay results. Moreover, standardization of any bioassay method is often difficult across sentinel sites because of variations in local temperature, humidity, and testing conditions, which significantly confound the bioassay results. Historically, knowledge of the mechanisms of resistance led to the identification of markers for resistance. Most of the markers used to for monitoring resistance to pyrethroids are DNA markers [14, 54, 57]. These DNA markers include the *kdr* mutation L1014S/F and an additional mutation in the *para* gene that was recently identified (N1575Y) [14, 54, 57]. Markers based on differential expression between resistant and susceptible mosquitoes (RNA markers) are emerging, primarily for metabolic detoxification genes [24]. The identification of RNA markers is technically challenged by 1) the limited stability of RNA and 2) the fact that genes known to be associated with insecticide resistance (i.e. metabolic detoxification genes and cuticular protein genes) belong to large gene families.

WHO guidelines define the resistant and susceptible phenotypes as that of mosquitoes alive or dead twenty-four hours after insecticide exposure, respectively [7]. Due to RNA degradation in dead specimens [58], a number of alternative strategies have been used to sample RNA from susceptible mosquitoes, including analyzing the expression profile of mosquitoes of susceptible populations/strains [21, 24, 50], field-caught unexposed mosquitoes [24] or approximating susceptibility with early knock-down [25, 28]. Each strategy has its advantages and disadvantages. For instance, susceptible laboratory strain mosquitoes usually do not share the same genetic background as field-caught mosquitoes and are expected to have lower genetic variability than field-caught mosquitoes. However, for the same reasons, there is less within-sample variation in the gene expression profiles of laboratory strains. Using field-caught mosquitoes has the advantage of limiting the impact of environmental conditions on a heterogeneous genetic background. However, unexposed field-caught mosquitoes are a mixture of resistant and susceptible individuals, and the expression profile of early knocked-down mosquitoes will include both constitutively expressed and insecticide-induced DE genes. As a consequence, a comparison across studies to find common RNA markers may be influenced by the chosen experimental design. Additionally, DE genes may have a direct effect on the resistance phenotype or an indirect or additive effect and may belong to large gene families [59, 60]. Gene products with functions such as metabolic detoxification, proteolysis and cuticular proteins have been linked to resistance to insecticides [14–21, 24, 25, 50, 53]. However, all of these proteins are members of large gene families, and it is unclear whether one/few members of each family have a major role in resistance [54] or whether functional convergence and additive effects contribute to the resistance phenotype. Functional convergence with respect to pyrethroid metabolism among the 111 annotated CYPs from the *An. gambiae* genome could explain variation across populations in CYP-encoding genes identified as significantly over-expressed in resistant mosquitoes through qRT-PCR, microarray and RNA-seq approaches [15, 21, 24, 25, 50, 54, 61]. If multiple CYPs are able to metabolize pyrethroids [16, 17], a mosquito can become resistant when certain CYPs act synergistically without invoking significant changes in the expression of individual CYPs. Additionally, some CYPs may show tissue-specific expression [62]. Alternatively, local adaptation may lead to over-expression of different CYPs in various geographic populations. From the operational standpoint of resistance monitoring, the observed variation across populations in the over-expression of CYP-encoding genes supports the possibility that sensitive and field-deployable biochemical assays that quantify overall monooxygenase activity [63, 64], may prove suitable for predicting the resistance status of a mosquito population [65].

### Candidate-resistance genes

Genes related to transcription and translation and response to stress were enriched among the 2457 genes that were DE between field-caught resistant and susceptible mosquitoes, suggesting that insecticide exposure is a stress for mosquitoes, leading to complex gene interaction mechanisms, including gene expression modulation. A comparison between these 2457 DE genes and the 182 constitutive DE genes found six detoxification genes constitutively and more highly expressed in resistant mosquitoes, including the previously identified CYPM2 and GSTE5 [15, 17, 24, 50], CYP303A1 (AGAP10077), CYP4C27 (AGAP009246), GSTD3 (AGAP004382) and HPX2. Fold-changes in differential expression ranged between 1.55 and 2.33, which suggests an additive effect on the resistant phenotype is more probable than a major effect by the product of one gene. An additive effect of multiple detoxification genes and/or functional convergence would explain the lack of detoxification genes among the 7 DE genes consistently and more highly expressed in resistant versus susceptible mosquitoes from Western Kenya in a span of 2 years (2010–2012).

Genes associated with proteolytic functions and cuticle appeared to be enriched among the constitutive genes expressed more in susceptible than resistant mosquitoes. Most of these cuticular protein genes were also more highly expressed in susceptible than resistance mosquitoes in 2010 [25], which is different than what was seen in *An. gambiae* and *An. coluzzii* mosquitoes from West Africa [24, 64]. Whether this result is related to the different ecosystem and more intense agricultural activities in West Africa in comparison to the Western Province of Kenya needs to be investigated. Areas near the equator in West and East Africa have a different seasonality. In West Africa, a long dry season is followed by a single intense rainy season between July and September [67]. In Kenya, two rainy seasons generally occur between April-August and November-December [67]. The longer dry season in West Africa may precondition mosquitoes with a thicker cuticle that could result in penetration resistance to insecticides.

When compared to a list of candidate-resistance genes recently identified from *An. coluzzii* mosquitoes from West Africa [24], 5 genes (AGAP004177, AGAP007530, AGAP008840, AGAP013036 and AGAP004572) showed similar significant differential expression, emphasizing their potential role as markers for resistance for closely related *An. gambiae/An. coluzzii* mosquitoes across different ecosystems. AGAP004177 and AGAP004572 are particularly interesting because they map within *rtp1*, a QTL previously associated with resistance to pyrethroid and including the *para* sodium channel gene [55]. AGAP004177 encodes a heat shock protein with 23S rRNA (uridine2552–2′-O)-methyltransferase activity [68]. Incorrect ribosomal methylation and/or impairment in ribosome maturation and function have been linked to drug resistance in prokaryotes [69, 70], suggesting the activity of this gene should be further investigated. AGAP004572 encodes a hypothetical protein with a fatty acid desaturase domain, based on the Eukaryotic Orthologous Groups (KOG) [71] and SMART databases [72]. Fatty acid desaturases are conserved proteins that create a double bond in long-chain fatty acids, the primary determinant of triglyceride melting temperature and cellular membrane fluidity [73]. Fatty acid desaturase activity and overexpression of cuticular proteins may provide an additional mechanism for insecticide penetration. AGAP013036 encodes a hypothetical protein, which the KOG database identifies as a probable phosphatidylethanolamine-binding protein (PEBP). PEBPs are highly conserved and have been associated with diverse functions such as lipid binding, signal transduction, serine protease inhibition and neuronal development [74], suggesting an indirect role in resistance. AGAP007530 encodes a hypothetical protein with a predicted zinc carboxypeptidase domain, based on KOG and SMART databases. In prokaryotes, zinc carboxypeptidases may be used to digest exogenous proteins and degrade tissues [75, 76], suggesting their potential role in insecticide catabolism should be investigated. AGAP008840 encodes a hypothetical protein linked to chromatin structure and dynamics. Its role in insecticide resistance is unclear.

### SNPs associated with the resistance phenotype

The *para* sodium channel is the target site for pyrethroid. Electrophysiological studies of this protein from *Drosophila melanogaster*, *Aedes aegypti*, *Musca domestica* and *Blatella germanica* expressed in *Xenopus* oocytes showed that the L1014F/S mutation alters its function and provides protection from pyrethroids [56]. The frequency of the L1014S/F mutation increased significantly in the past 10 years across sub-Saharan Africa in both *An. gambiae* and *An. coluzzii*, concomitantly with the implementation of malaria control strategies [8, 9]. This coincidence was used to support the conclusion that there is causal relationship between L1014S/F mutations at the *para* sodium channel and the resistant phenotype [56]. In our experiments, RNA-seq data and genotyping in individually-phenotyped mosquitoes showed the L1014S mutation in both insecticide-resistant and susceptible *An. gambiae* mosquitoes, confirming that the L1014S mutation alone cannot fully account for the resistant phenotype [77]. Recent genotyping data showed that the L1014F does not account for high levels of resistance in *An. coluzzii* mosquitoes from Burkina Faso [24] nor it is linked with the resistant phenotype in *An. gambiae* and *An. coluzzii* mosquitoes from Nigeria and Benin [64, 66]. These results suggest that additional mechanisms of resistance have evolved, including novel mutations, such as N1575Y [14], and/or changes to gene expression. For instance, if metabolic detoxification mechanisms or penetration resistance are prevailing [66], the amount of insecticide available to bind the insecticide target will be reduced, explaining the observed limited correlation between L1014F/S and the resistant phenotype [14, 66]. In this scenario, the difference between resistant and susceptible mosquitoes could result from several possibilities, including barriers to insecticides penetration, catabolic pathways employed by resistant and susceptible mosquitoes, secondary pathways involved in the metabolism of insecticide intermediates and/or a more efficient “pyrethrome” [78] in resistant mosquitoes resulting in a quicker insecticide degradation and/or sequestration and elimination of toxic secondary metabolites.

RNA-seq data, followed by genotyping on singly phenotyped mosquitoes, identified the mutation A101G in the gene AGAP009551, which was associated with the resistant phenotype and should be further investigated. AGAP009551 is annotated as a sulfotransferase and maps within *rtp3* [55]. Sulfotransferases catalyze the sulfate conjugation of hormones and xenobiotic compounds in humans and plants [79]. Sulfonation can lead to either enhanced secretion of the compounds or the production of bioactive metabolites [79]. Primary catabolic pathways identified for deltamethrin include the CYP-mediated hydroxylation of deltamethrin to 4′-hydroxydeltamthrin [17]; the final metabolites in the pathway include trans-hydroxymethyl deltamethrin and deltamethric acid. Alternatively, cleavage of the ester bond by carboxylesterases renders the fragment cyclopropane carboxylic acid and phenoxybenzylic alcohol inactive as insecticides [79]. Sulfotransferase-mediated conjugation could be an alternative degradation route for deltamehtrin, or act on the metabolites produced after oxidation, reduction or hydrolysis in Phase I of the degradation process. Sulfotransferases target electrophilic centers favoring conjugation to cellular sugars, glutathione or amino acids (biotransformation, Phase II of the degradation process) [12].

## Conclusions

By comparing the expressional profile of deltamethrin-resistant and –susceptible mosquitoes from Western Kenya, we identified five genes with a similar expression profile in resistant *An. gambiae* and *An. coluzzii* mosquitoes from West and East Africa, suggesting these genes could be used as expression-based markers for resistance. We also identified a SNP in the sulfotraferase gene AGAP009551 that is strongly associated with insecticide resistance. No correlation with the resistant phenotype was seen for the *kdr* mutation L1014S.

Overall, our results support the idea that many genes may be involved synergistically in insecticide resistance, with their role being modulated by the life history and selection pressure of mosquito populations.

## Additional files

Additional file 1: List of primers. List of genes/primers used in qRT-PCR (A) and in genotyping (B). (XLSX 15 kb) Additional file 2:
**Quality control of** **RNA-seq** **data: Box Plot of transcript quantification levels.** The distributions of FPKM scores across samples are visualized. (PDF 130 kb) Additional file 3:
**Output of the Tuxedo pipeline on gene differential expression.** List of genes analyzed, gene quantification levels (output of Cufflinks) and analyses of differential expression (output of Cuffdiff). (XLSX 6499 kb) Additional file 4:
**Lists of DE genes.** List of the 2457 genes significantly DE between field-caught resistant and susceptible mosquitoes (Sheet 2457 RvS). List of the 182 genes significantly DE between field-caught resistant and susceptible mosquitoes of the Kisumu strain, but not field-caught susceptible mosquitoes and Kisumu mosquitoes (sheet 182 constitutive DE gene). List of the 55 DE genes in mosquitoes from Western Kenya in 2010 and 2012 (Sheet 55 candidate resistance genes). (XLSX 394 kb) Additional file 5:
**Detoxification genes.** Fold changes in gene expression across detoxification-related genes. Genes previously associated with insecticide resistance are marked with a red arrow. (PDF 173 kb) Additional file 6:
**Candidate-resistance genes.** List of candidate resistance genes detected in *An. gambiae* mosquitoes from West and East Africa. (XLSX 18 kb) Additional file 7:
**qRT-PCR on candidate resistance genes.** The level of expression of 4 candidate resistance genes was measured by qRT-PCR from different resistant and susceptible mosquitoes than those used for RNA-seq. All tested genes were significantly differentially expressed between resistant and susceptible mosquitoes. (PDF 35 kb) Additional file 8:
**Novel SNP in the** ***para*** **gene.** Visualization of the change from A to G that was identified at nucleotide position 2391280 of the *para* gene (AGAP004707) from RNA-seq data. Gray horizontal lines represent RNA-seq reads from libraries of both resistant and susceptible mosquitoes. (PDF 17 kb) Additional file 9:
**SNP-genes. **List of genes with SNPs identified only in resistant mosquitoes from RNA-seq data and Odd ratio analyses for the genes that were genotyped in single mosquitoe. (XLSX 49 kb)
