# Supplementary figures and images for: RNA-seq analyses of changes in the Anopheles gambiae transcriptome associated with resistance to pyrethroids in Kenya: identification of candidate-resistance genes and candidate-resistance SNPs
Source: Parasit Vectors. 2015 Sep 17;8:474. doi: 10.1186/s13071-015-1083-z (PMC4574070; doi:10.1186/s13071-015-1083-z)

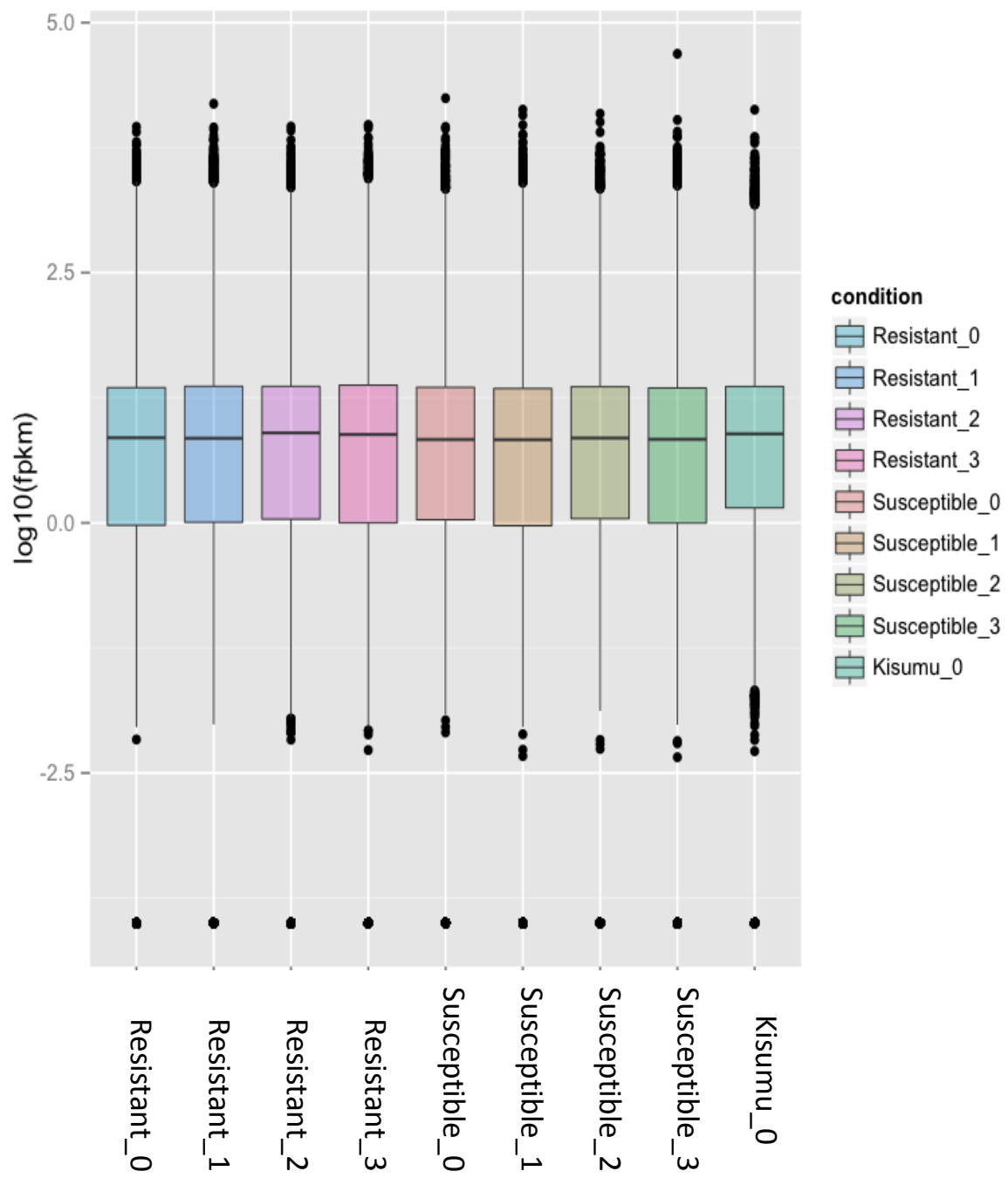

Supplement: Additional file 2: — Quality control of RNA-seq data: Box Plot of transcript quantification levels. The distributions of FPKM scores across samples are visualized. (PDF 130 kb) [file 13071_2015_1083_MOESM2_ESM.pdf]

R/S

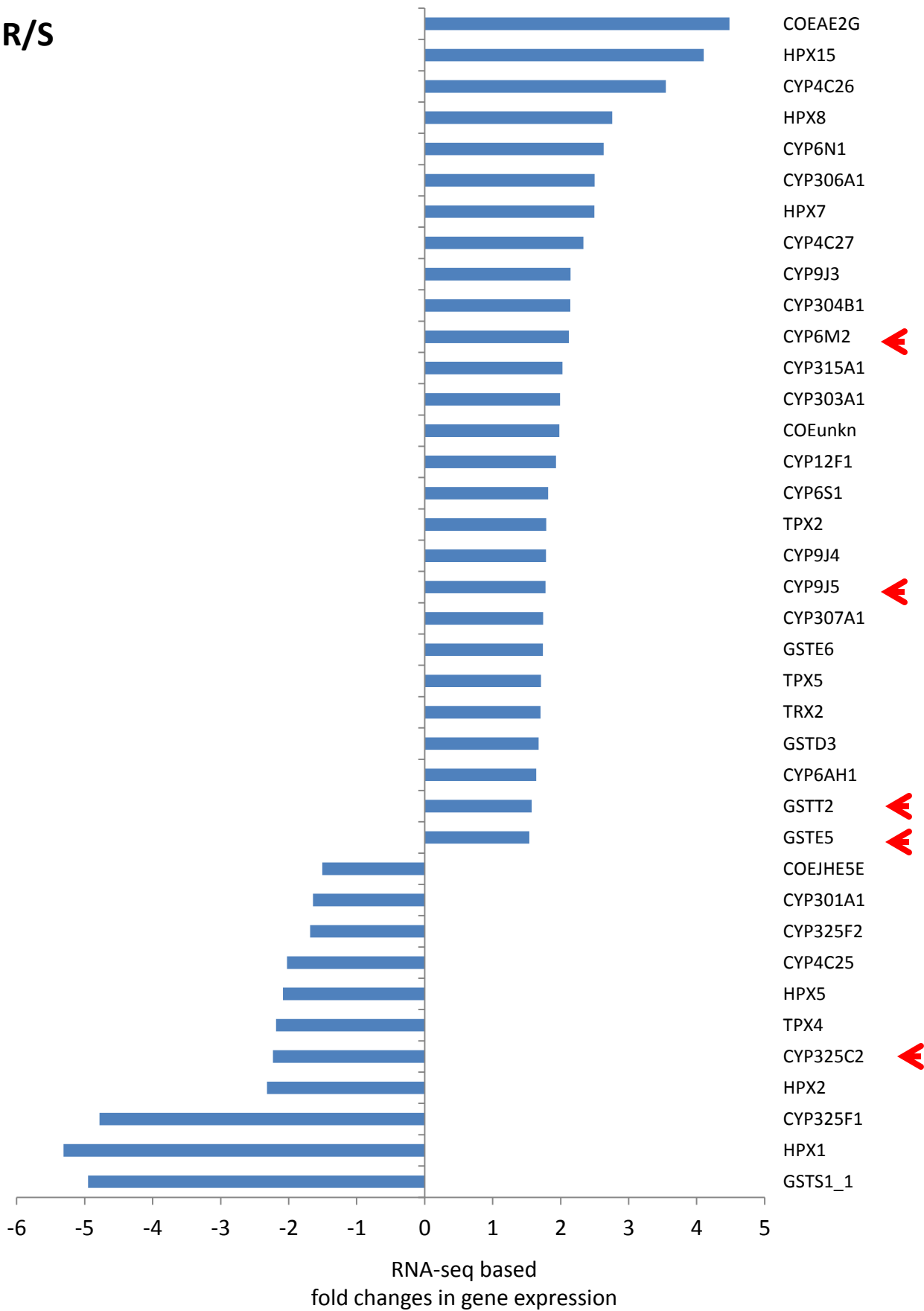

Supplement: Additional file 5: — Detoxification genes. Fold changes in gene expression across detoxification-related genes. Genes previously associated with insecticide resistance are marked with a red arrow. (PDF 173 kb) [file 13071_2015_1083_MOESM5_ESM.pdf]

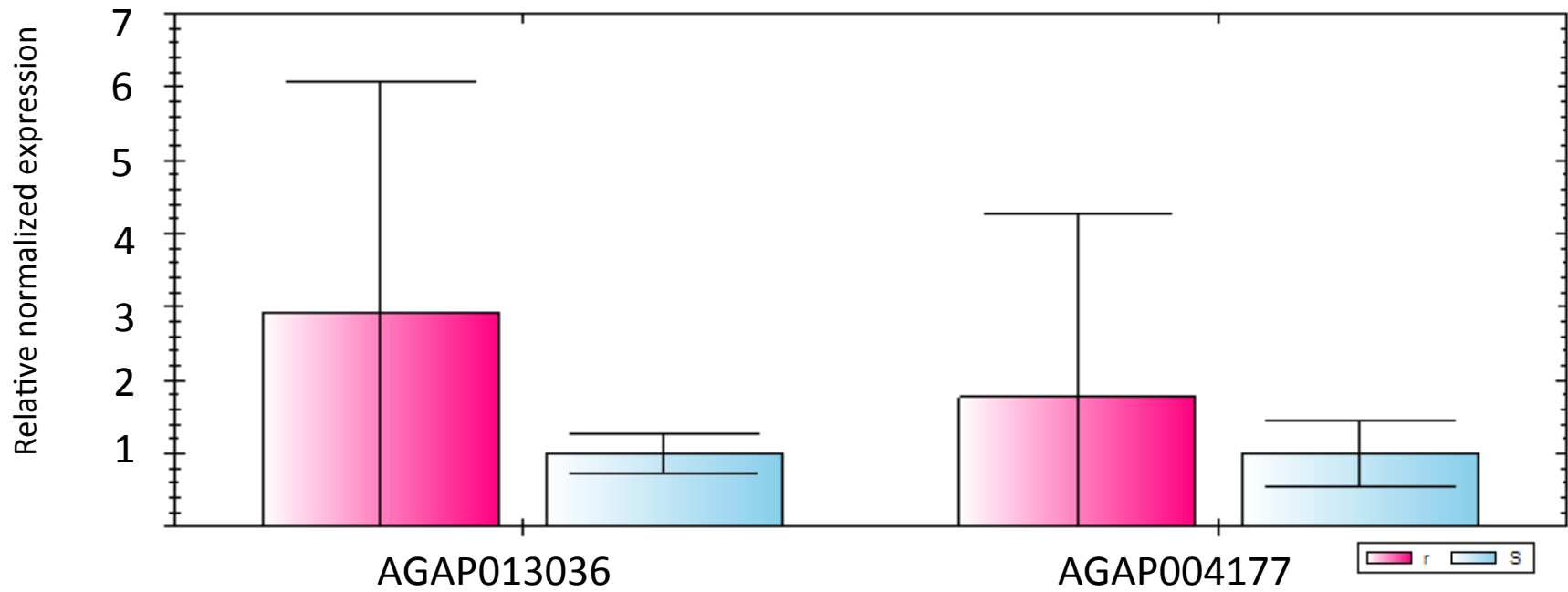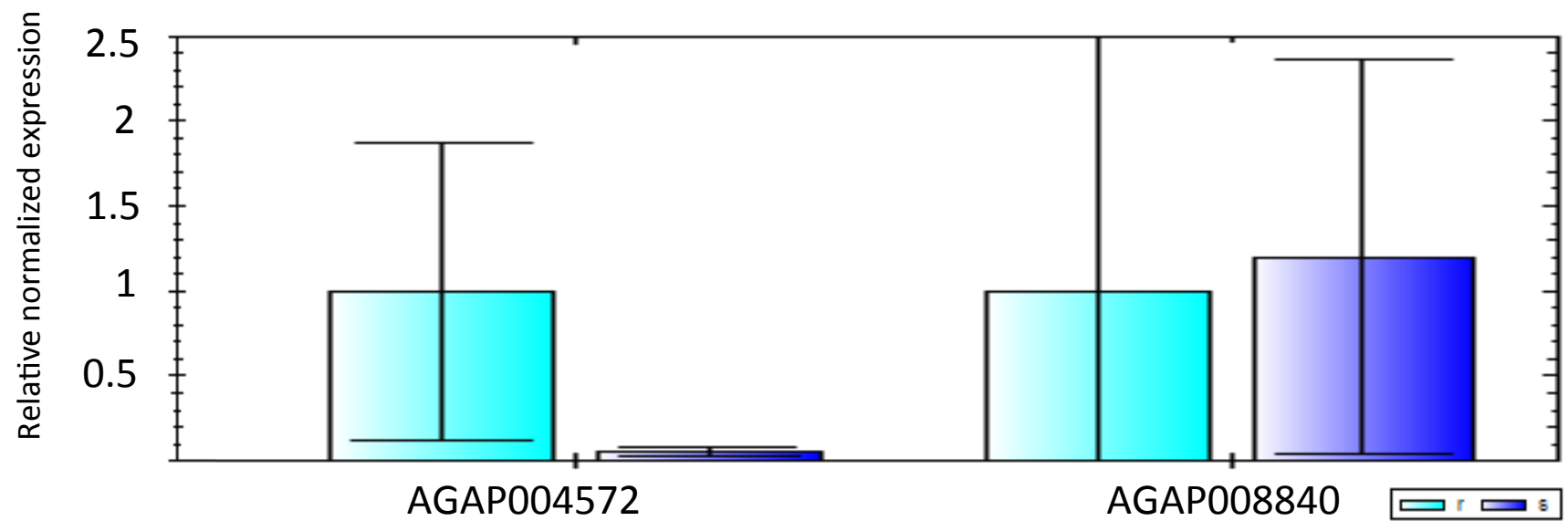

Supplement: Additional file 7: — qRT-PCR on candidate resistance genes. The level of expression of 4 candidate resistance genes was measured by qRT-PCR from different resistant and susceptible mosquitoes than those used for RNA-seq. All tested genes were significantly differentially expressed between resistant and susceptible mosquitoes. (PDF 35 kb) [file 13071_2015_1083_MOESM7_ESM.pdf]

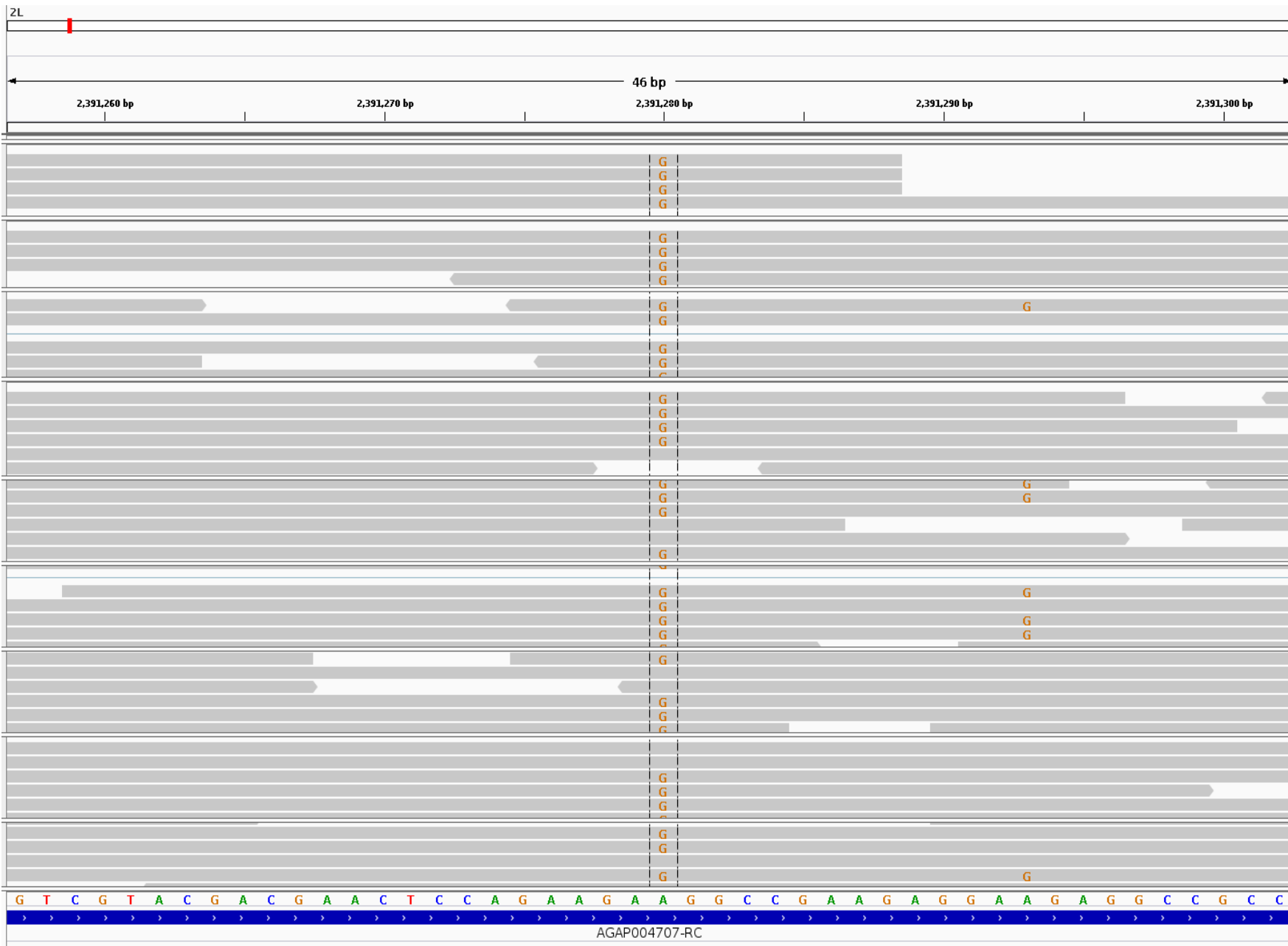

Supplement: Additional file 8: — Novel SNP in the para gene. Visualization of the change from A to G that was identified at nucleotide position 2391280 of the para gene (AGAP004707) from RNA-seq data. Gray horizontal lines represent RNA-seq reads from libraries of both resistant and susceptible mosquitoes. (PDF 17 kb) [file 13071_2015_1083_MOESM8_ESM.pdf]
